# Changes in serum albumin concentrations over 7 days in medical inpatients with and without nutritional support. A secondary post-hoc analysis of a randomized clinical trial

**DOI:** 10.1038/s41430-023-01303-w

**Published:** 2023-07-07

**Authors:** Fabienne Boesiger, Alessia Poggioli, Claudine Netzhammer, Céline Bretscher, Nina Kaegi-Braun, Pascal Tribolet, Carla Wunderle, Alexander Kutz, Dileep N. Lobo, Zeno Stanga, Beat Mueller, Philipp Schuetz

**Affiliations:** 1 Medical University Department, Division of General Internal and Emergency Medicine, Kantonsspital Aarau, Aarau, Switzerland; 2 Department of Health Professions, Bern University of Applied Sciences, Bern, Switzerland; 3 Faculty of Life Sciences, University of Vienna, Vienna, Austria; 4 Gastrointestinal Surgery, Nottingham Digestive Diseases Centre and National Institute for Health Research (NIHR) Nottingham Biomedical Research Centre, Nottingham University Hospitals NHS Trust and University of Nottingham, Queen’s Medical Centre, Nottingham, UK; 5 MRC Versus Arthritis Centre for Musculoskeletal Ageing Research, School of Life Sciences, University of Nottingham, Queen’s Medical Centre, Nottingham, UK; 6 Division of Diabetes, Endocrinology, Nutritional Medicine, and Metabolism, Inselspital, Bern University Hospital, University of Bern, Bern, Switzerland; 7 Department of Clinical Research, University Hospital Basel, University of Basel, Basel, Switzerland

**Keywords:** Nutrition, Biomarkers

## Abstract

**Background:**

Serum albumin concentrations are frequently used to monitor nutritional therapy in the hospital setting but supporting studies are largely lacking. Within this secondary analysis of a randomized nutritional trial (EFFORT), we assessed whether nutritional support affects short-term changes in serum albumin concentrations and whether an increase in albumin concentration has prognostic implications regarding clinical outcome and response to treatment.

**Methods:**

We analyzed patients with available serum albumin concentrations at baseline and day 7 included in EFFORT, a Swiss-wide multicenter randomized clinical trial that compared individualized nutritional therapy with usual hospital food (control group).

**Results:**

Albumin concentrations increased in 320 of 763 (41.9%) included patients (mean age 73.3 years (SD ± 12.9), 53.6% males) with no difference between patients receiving nutritional support and controls. Compared with patients that showed a decrease in albumin concentrations over 7 days, those with an increase had a lower 180-day mortality [74/320 (23.1%) *vs*. 158/443 (35.7%); adjusted odds ratio 0.63, 95% CI 0.44 to 0.90; *p* = 0.012] and a shorter length of hospital stay [11.2 ± 7.3 *vs*. 8.8 ± 5.6 days, adjusted difference −2.2 days (95%CI −3.1 to −1.2)]. Patients with and without a decrease over 7 days had a similar response to nutritional support.

**Conclusion:**

Results from this secondary analysis indicate that nutritional support did not increase short-term concentrations of albumin over 7 days, and changes in albumin did not correlate with response to nutritional interventions. However, an increase in albumin concentrations possibly mirroring resolution of inflammation was associated with better clinical outcomes. Repeated in-hospital albumin measurements in the short-term is, thus, not indicated for monitoring of patients receiving nutritional support but provides prognostic information.

**Trail Registration:**

ClinicalTrials.gov Identifier: NCT02517476.

## Introduction

Historically, serum albumin concentration was considered to be a marker of nutritional status and physicians monitored albumin concentrations in patients during their hospital stay. This assumption was based on the pathophysiological grounds that albumin concentration reflects circulating proteins in plasma, with lower concentrations indicating nutritional deficiencies [1, 2]. However, it has been recognized for years that albumin as well as other visceral proteins (e.g., prealbumin) are markers of inflammation and correlate negatively with the severity of acute illness, but show little correlation with nutritional status [3–7]. In fact, albumin should be considered a negative acute-phase-protein, with concentrations declining in acute and chronic illness due to hepatic reprioritization of protein synthesis [2, 8, 9] and increased transcapillary escape of albumin [10]. In addition, albumin concentrations can fluctuate with hydration status [11, 12]. Yet, in clinical practice, many physicians still continue to monitor albumin concentrations to evaluate the response to nutritional support although supporting studies have been lacking [13].

Malnutrition is a common condition among medical inpatients, with a prevalence of about 30%, and is associated with increased mortality, morbidity, disability, and higher health care costs [14–19]. Several studies have shown that nutritional support reduces mortality as well as other adverse outcomes [19–21]. Therefore, it is important to early identify patients who are nutritionally at risk and provide them with appropriate nutritional therapy to reduce risks for clinical deterioration and, perhaps, improve outcomes. Nutritional screening based on a validated screening tool is the first step to identify patients at risk of malnutrition [17, 18, 22]. While screening tools are sensitive for diagnosis of malnutrition, they may not predict response to treatment [23]. More specific clinical parameters and blood biomarkers are needed to allow a more personalized approach to malnourished patients as not all patients show the same response to nutritional interventions. Recent studies have suggested that some nutritional biomarkers of inflammation, kidney function and muscle health, among others, predict treatment response to nutritional interventions and may help to personalize treatments. [14, 24–27] We recently found albumin concentrations measured in patients at hospital admission to be helpful to predict clinical outcomes among patients at nutritional risk, but albumin was not helpful in predicting treatment response to nutritional intervention [3]. Also, in the same trial, we measured prealbumin levels on admission, which has a shorter half-life as compared with albumin, but still only provided little information regarding nutritional treatment response [28]. In addition to baseline levels of these visceral proteins at hospital admission, there is still insufficient evidence regarding the usefulness of short-term changes in albumin concentrations over time to predict treatment response [2].

Herein, we tested the hypothesis that nutritional support influences short-term changes in serum albumin concentrations in medical inpatients and that these changes would correlate with medical outcomes and response to nutritional support in patients included in the Effect of early nutritional therapy on Frailty, Functional Outcomes, and Recovery on malnourished medical inpatients Trial (EFFORT) [19].

## Material & Methods

### Study design and setting

This is a secondary analysis of EFFORT [19], a pragmatic, multicenter, open-label, investigator-initiated trial performed in 8 Swiss hospitals from April 2014 to February 2018. The trial investigated the effect of early nutritional support versus standard hospital food on patient outcomes in medical inpatients. The study protocol was approved by the Ethics Committee of Northwestern Switzerland (EKNZ; 2014_001). All participants, or their authorized representatives, provided written informed consent. EFFORT was registered at ClinicalTrials.gov (https://clinicaltrials.gov/ct2/show/NCT02517476). Detailed information about rationale, design as well as the results of the trial have been published elsewhere [19, 29].

### Patient population and management

EFFORT enrolled adult ( ≥ 18 years of age) medical inpatients at nutritional risk with an anticipated hospital stay of at least 5 days who were willing to give informed consent within the first 48 hours after admission. Nutritional risk was defined as a Nutritional Risk Screening (NRS 2002) score of 3 points or more. The NRS 2002 consists of two parts: the patient’s current nutritional status and the severity of the underlying disease. Both parts score from 0 (absent) to 3 (severe) with an extra point for age ≥ 70 years. An total score of 3 or more points indicates “nutritionally at risk” and additional nutritional support should be considered [18, 30, 31]. More detailed information about NRS 2002 is provided in the Supplement. Patients were excluded if they were initially admitted to intensive care or surgical units, were incapable of ingesting food orally, had contraindications to nutritional supplements, were already receiving nutritional support at admission, were previously included in the study, had a terminal condition, anorexia nervosa, acute pancreatitis, acute liver failure, cystic fibrosis, stem cell transplantation or bariatric surgery. Participants were randomly assigned (1:1) by an interactive web-system to receive either individual nutritional therapy (intervention group) or standard hospital food (control group). As for the intervention group, individualized nutritional support was established within the first 48 h after admission. Energy and protein goals were calculated by a trained nutritionist who then developed an individual treatment plan for each patient. The initial approach was to use nutritional support by the oral route. If patients did not reach 75% of their protein and energy goals within 5 days, therapy could be escalated to enteral tube or parenteral feeding. Upon admission, several other parameters including Barthel’s index [32] were collected according the trial protocol. Trained study nurses conducted a structured telephone interview to systematically assess predefined health-related outcomes 30 and 180 days after discharge.

### Research objective and outcomes

We had three main goals for this analysis: first, to investigate how nutritional therapy impacts on the short-term changes in serum albumin concentrations from baseline to 7 days in the overall population and within subgroups of patients with high and low baseline albumin concentrations ( < 30 g/L or ≥ 30 g/L) [3, 33]. We used the same cut-offs for albumin as used in our previous publication [3] and which corresponds to normal values based on the assay used for measurement. Second, we aimed to investigate whether changes in serum albumin concentrations would predict clinical and functional outcomes and, thirdly, response to nutritional support. To investigate these hypotheses, we calculated several models in the overall population and further stratified patients based on their inflammatory status, i.e., based on their levels of CRP similar to a previous analysis [27]. Stratification was done low CRP ( < 100 mg/l) and high CRP ( ≥ 100 mg/l) on admission.

Our primary endpoint for the prognostic analyses was long-term all-cause mortality measured over 180 days, while for the response to nutritional support we focused on short-term 30-day mortality. Secondary endpoints where adverse outcome within 30 days (composite endpoint consisting of all-cause mortality, admission to the intensive care unit from medical ward, major complications (nosocomial infection, respiratory failure, major cardiovascular event, acute renal failure, gastrointestinal failure), nonelective hospital readmission after discharge, decline in functional status ≥10% measured by Barthel’s index), length of hospital stay, loss of function according to Barthel’s index (score ranging from 0 to 100 with lower scores indicating worse functional status) and quality of life measured by 5-level European Quality of life 5 Dimensions index (EQ5D) including the self-assessment visual analogue scale (VAS). Detailed information for the single endpoints and their composites is summarized in the Supplement. We defined treatment response as the difference in outcomes among control group and intervention group patients, similar to the initial EFFORT trial.

### Statistical analyses

Continuous variables are shown as means and standard deviation. Categorical and binary data are expressed as counts and percentages. Baseline characteristics were compared between patients with an increase in albumin and without an increase in albumin after 7 days using Pearson’s χ^2^ test for binary and categorical variables and Student t-test for continuous variables. We also studied the association of an increase in serum albumin with different clinical outcomes in regression analysis. We used logistic regression with odds ratios (OR) and 95% confidence interval (CI) for binary outcomes and linear regression with coefficient (Coef) and 95% confidence interval (CI) for continuous variables. All analyses were adjusted for the following predefined covariates: age, sex, main diagnosis, comorbidities and study center. Statistical analyses were performed with STATA 15.1 (Stata Corp, College Station, TX, USA).

## Results

### Patient population

**Fig. 1:**
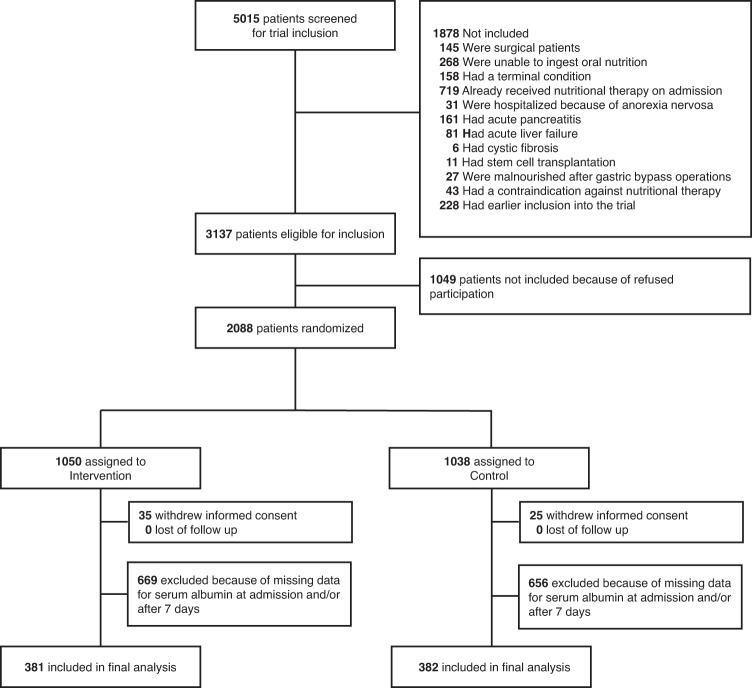
Study flow. Of 5015 patients initially screened, 2088 were randomized and 381 patients included in the intervention group and 382 in the control group.

From the 2088 participants of the initial trial, we had complete data on 763 patients regarding baseline and day 7 albumin concentration and all clinical outcomes (Fig. 1). Mean age was 73 ( ± 13) years and 54% of participants were male. A total of 320 participants showed an increase in serum albumin concentration after 7 days (delta albumin from baseline to day 7), while 443 showed a decrease. Patients with albumin increase had a higher body mass index on admission and had differences regarding the main admission diagnosis and types of comorbidities compared with patients with no albumin increase. Table 1 shows baseline characteristics for the overall trial cohort and stratified by increase or decrease in albumin after 7 days.

**Table 1:** Baseline characteristics overall and stratified according to increase in albumin over 7 days.

	Overall	Increase in albumin after 7 days	Decrease in albumin after 7 days	*p*-value
	763	320	443	
**S**ociodemographics				
Age, mean (SD) years	73.3 (12.9)	73.2 (13.6)	73.3 (12.4)	0.92
Male sex	409 (53.6%)	164 (51.2%)	245 (55.3%)	0.27
Nutritional assessment				
BMI, mean (SD) kg/m^2^	24.9 (5.3)	25.3 (5.5)	24.5 (5.1)	0.048
Weight at admission, mean (SD) kg	72.0 (16.3)	73.1 (17.2)	71.1 (15.7)	0.15
Height, mean (SD) cm	168.1 (8.9)	167.6 (9.3)	168.5 (8.6)	0.17
NRS 2002 score				
3 points	203 (26.6%)	86 (26.9%)	117 (26.4%)	0.73
4 points	305 (40.0%)	128 (40.0%)	177 (40.0%)	
5 points	208 (27.3%)	83 (25.9%)	125 (28.2%)	
6 points	47 (6.2%)	23 (7.2%)	24 (5.4%)	
Markers of inflammation				
CRP (mg/dl), mean (SD)	87 (91)	106 (98)	56 (71)	0.048
Admission diagnosis				
Infection	217 (28.4%)	119 (37.2%)	98 (22.1%)	<0.001
Cancer	180 (23.6%)	54 (16.9%)	126 (28.4%)	<0.001
Cardiovascular disease	89 (11.7%)	41 (12.8%)	48 (10.8%)	0.40
Failure to thrive	52 (6.8%)	14 (4.4%)	38 (8.6%)	0.023
Lung disease	40 (5.2%)	17 (5.3%)	23 (5.2%)	0.94
Gastrointestinal disease	62 (8.1%)	22 (6.9%)	40 (9.0%)	0.28
Neurological disease	14 (1.8%)	7 (2.2%)	7 (1.6%)	0.54
Renal disease	39 (5.1%)	13 (4.1%)	26 (5.9%)	0.26
Metabolic disease	28 (3.7%)	12 (3.8%)	16 (3.6%)	0.92
Other	25 (3.3%)	13 (4.1%)	12 (2.7%)	0.30
Comorbidities				
Hypertension	437 (57.3%)	188 (58.8%)	249 (56.2%)	0.48
Malignant disease	287 (37.6%)	107 (33.4%)	180 (40.6%)	0.043
Chronic kidney disease	268 (35.1%)	126 (39.4%)	142 (32.1%)	0.037
Coronary heart disease	184 (24.1%)	77 (24.1%)	107 (24.2%)	0.98
Diabetes mellitus	179 (23.5%)	67 (20.9%)	112 (25.3%)	0.16
Congestive heart failure	145 (19.0%)	70 (21.9%)	75 (16.9%)	0.086
Chronic obstructive pulmonary disease	93 (12.2%)	41 (12.8%)	52 (11.7%)	0.65
Peripheral arterial disease	73 (9.6%)	31 (9.7%)	42 (9.5%)	0.92
Cerebrovascular disease	70 (9.2%)	28 (8.8%)	42 (9.5%)	0.73
Dementia	23 (3.0%)	9 (2.8%)	14 (3.2%)	0.78

*CRP* C-reactive protein, *BMI* Body mass index, *NRS* Nutritional risk screening, *SD* Standard deviation.

An additional stratification by low C-reactive protein (CRP) ( < 100 mg/l) and high CRP ( ≥ 100 mg/l) on admission is provided in the Supplement (Table 1).

### Change in albumin concentration from baseline to day 7 in patients with and without nutritional intervention

Table 2 shows albumin concentrations at baseline and day 7 in patients with and without nutritional support. Mean serum albumin concentration in the control group at baseline was 27.9 g/L and dropped slightly by 0.72 g/L to 27.2 g/L. Results in the nutritional intervention group were similar with a drop from 27.7 g/L to 26.9 g/L. There was no difference between intervention and control group patients regarding changes in albumin concentration [mean difference −0.06 g/L (95%CI −0.55 to 0.44)]. A corresponding analysis stratified for normal or low baseline albumin concentrations was similar but there was a slight higher proportion of patients with an albumin increase in the control group (81% vs. 63.3%, *p* = 0.002), a finding that remained significant in the adjusted regression analysis. We also performed additional stratification by low and high baseline CRP concentrations, which again showed similar results, but in patients with higher inflammation there was a stronger increase in albumin over time without differences among treatment groups (Supplement Tables 2.1, 2.2).

**Table 2:** Changes in serum albumin concentrations from baseline to day 7 in with and without nutritional support overall, and according to baseline albumin levels.

	No nutritional support (Control)	Nutritional support (Intervention)	*p*-value	Adjusted difference^a^ OR or Coef (CI 95%)	*p*-value
**A) All patients**					
**Change in albumin over 7 days**, mean (SD)					
• Baseline albumin (g/L)	27.93 (6.04)	27.67 (5.34)			
• Albumin after 7 days (g/L)	27.21 (5.41)	26.89 (5.42)			
• Change in albumin (g/L),	−0.72 (−1.07 to −0.37)	−0.78 (−1.12 to −0.43)	0.822	0.0 (−0.48 to 0.48)	0.991
**Increase** **vs. decrease in albumin after 7 days**					
• Patients with increase, *n*(%)	225/382 (58.9%)	218/381 (57.2%)			
• Patients with decrease, *n*(%)	157/382 (41.1%)	163/381 (42.8%)	0.638	1.11 (0.82–1.5)	0.488
**B) Subgroup analysis: Baseline-Albumin** **<** **30** **g/l**					
**Change in albumin after 7 days**, mean (SD)					
• Baseline albumin (g/L)	24.34 (3.75)	24.87 (3.55)			
• Albumin after 7 days (g/L)	24.71 (4.48)	24.59 (4.43)			
• Change in albumin (g/L)	0.37 (−0.02 to 0.76)	−0.28 (−0.66–0.1)	**0.018**	−0.48 (−1 to 0.05)	0.074
**Increase** **vs. decrease in albumin after 7 days**					
• Patients with increase, *n*(%)	114/245 (46.5%)	142/261 (54.4%)			
• Patients with decrease, *n*(%)	131/245 (53.5%)	119/261 (45.6%)	0.077	0.76 (0.53–1.1)	0.146
**C) Subgroup analysis: Baseline-Albumin** **>** **30** **g/l**					
**Change in albumin after 7 days**, mean (SD)					
• Baseline albumin (g/L)	34.35 (3.48)	33.75 (2.99)			
• Albumin after 7 days (g/L)	31.68 (3.83)	31.89 (3.77)			
• Change in albumin (g/l)	−2.67 (−3.24 to −2.1)	−1.86 (−2.56 to −1.17)	0.073	0.82 (−0.08 to 1.72)	0.074
**Increase** **vs. decrease in albumin after 7 days**					
• Patients with increase, *n*(%)	111/137 (81%)	76/120 (63.3%)			
• Patients with decrease, *n*(%)	26/137 (19.0%)	44/120 (36.7%)	**0.002**	2.43 (1.34–4.42)	**0.004**

*SD* Standard deviation, *CI 95%* Confidence interval, *OR* Odds ratio, *Coef.* Coefficient.

^a^Adjusted for age, sex, main diagnosis, comorbidities and study center.

Bold values indicates statistical significant *P*-values (*P* < 0.05).

### Association of kinetics of serum albumin level and clinical outcomes

**Fig. 2:**
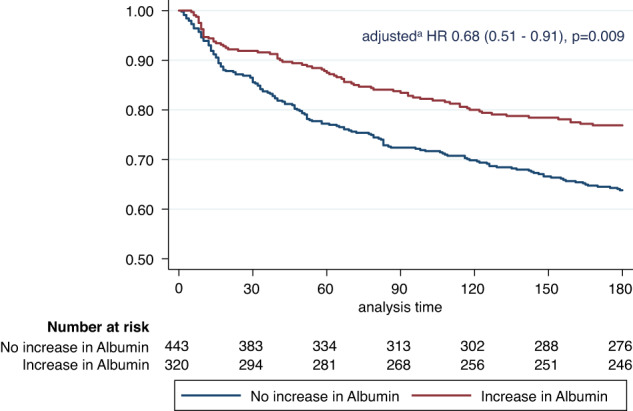
Survival of patients according to increase or decrease in albumin levels over 7 days. Kaplan–Meier estimate for 180-days mortality for increase and decrease in serum albumin from baseline to day 7.

We then investigated the prognostic value of changes in albumin concentrations regarding different clinical and functional outcomes (Table 3). Overall, the short-term changes in albumin concentrations were highly predictive for different short- and long-term clinical outcomes of patients. Participants with an increase in albumin showed a significantly reduced 180-days mortality [74/320 (23.1%) *vs*. 158/443 (35.7%); adjusted OR 0.63, 95% CI 0.44 to 0.9; *p* = 0.012)] and a reduced length of hospital stay (8.8 days *vs*. 11.16 days; adjusted difference −2.16 days, 95% CI −3.14 to 1.18; *p* < 0.001)]. Figure 2 shows the Kaplan-Meier-estimate for all-cause mortality within 180 days. When additionally stratifying by CRP concentrations, most results remained robust except for mortality in the low CRP group (Supplement Table 3.1–3.3).

**Table 3:** Clinical and functional outcomes in patients with and without an increase in albumin over 7 days.

	Overall			CRP < 100 mg/L			CRP ≥ 100 mg/L		
	events	adjusted ^a^		events	adjusted ^a^		events	adjusted ^a^	
	*n* (%) or mean (SD)	OR or Coeff (95% CI)	*p*-value	*n* (%) or mean (SD)	OR or Coeff (95% CI)	*p*-value	*n* (%) or mean (SD)	OR or Coeff (95% CI)	*p*-value
**Primary endpoints**									
**30-day mortality**									
Decrease in albumin	61/443 (13.8)	reference		35/298 (11.7)	reference		26/145 (17.9)	reference	
Increase in albumin	26/320 (8.1)	0.61 (0.37–1.02)	0.061	13/213 (6.1)	0.53 (0.26–1.07)	0.077	13/107 (12.2)	0.71 (0.32–1.56)	0.394
**180-day mortality**									
Decrease in albumin, *n*(%)	158/443 (35.7%)	reference		101/298 (33.9%)	reference		57/145 (39.3%)	reference	
Increase in albumin, *n*(%)	74/320 (23.1%)	0.63 (0.44–0.9)	**0.012**	49/213 (23%)	0.68 (0.43–1.07)	0.096	25/107 (23.4%)	0.5 (0.26–0.96)	**0.037**
**Secondary endpoints**									
**Adverse outcome within 30 days**									
Decrease in albumin	138/443 (31.2)	reference		90/298 (30.2)	reference		48/145 (33.1)	reference	
Increase in albumin	75/320 (23.4)	0.72 (0.51–1.02)	0.068	47/213 (22.1)	0.66 (0.42–1.02)	0.061	28/107 (26.2)	0.8 (0.44–1.46)	0.472
**Length of hospital stay**									
Decrease in albumin, *n*(%)	11.16 (7.3%)	reference		10.57(6.8%)	reference		12.35(8.3%)	reference	
Increase in albumin, *n*(%)	8.8 (5.6%)	−2.16 (−3.14 to −1.18)	**<** **0.001**	8.93(5.7%)	−1.35 (−2.5 to −0.2)	**0.022**	8.55(5.4%)	−3.62 (−5.47 to 1.77)	**<** **0.001**
**Loss of function (Barthel index)**									
Decrease in albumin	83/443 (18.7)	reference		53/298 (17.8)	reference		30/145 (20.7)	reference	
Increase in albumin	39/320 (12.2)	0.66 (0.42–1.02)	0.058	22/213 (10.3)	0.54 (0.31–0.96)	**0.036**	17/107 (15.9)	0.86 (0.42–1.76)	0.674
**Quality of life (EQ5D-VAS)**									
Decrease in albumin, *n*(%)	68 (19.7%)	reference		68.17 (19.7%)	reference		67.6 (19.8%)	reference	
Increase in albumin, *n*(%)	68.01 (19.6%)	−0.43 (−4.1–3.24)	0.817	65.97 (18.7%)	−2.8 (−7.2–1.6)	0.211	72.25 (20.8%)	5.28 (−1.44 to 12)	0.123

*SD* Standard deviation, *CI 95%* Confidence interval, *OR* Odds ratio, *Coeff* Coefficient.

^a^Adjusted for age, sex, main diagnosis, comorbidities and study center.

Bold values indicates statistical significant *P-*values (*P* < 0.05).

### Predictors for changes in albumin concentrations from baseline to day 7

Further, we investigated which factors were associated with an increase in albumin concentrations from baseline to day 7 in a linear regression analysis (Table 4). In the univariate model, we found several factors that were associated with an increase in albumin including higher protein and energy intake, and different admission diagnoses. When adjusting for baseline albumin and CRP concentrations, those results remained robust.

**Table 4:** Univariate and multivariate regression models: Predictors for a change in albumin from baseline to day 7.

Parameters	univariate		adjusted for baseline albumin and CRP	
	Coeff (95% CI)	*p*-value	Coeff (95% CI)	*p*-value
**Sociodemographics**				
Age	0.14 (−0.2 to 0.48)	0.413	0.09 (−0.22 to 0.4)	0.568
Male sex	−0.2 (−0.69 to 0.3)	0.437	−0.44 (−0.89 to 0.01)	0.057
**Nutritional assessment**				
BMI < 18.0 kg/m^2^	reference		reference	
BMI 18.0–24.9 kg/m^2^	0.47 (−0.79 to 1.74)	0.462	0.49 (−0.66–1.64)	0.399
BMI ≥ 25.0 kg/m^2^	1.03 (−0.24 to 2.3)	0.111	1.15 (−0.01–2.3)	0.052
NRS 2002 score 3	reference		reference	
NRS 2002 score 4	−0.2 (−0.81 to 0.42)	0.526	−0.44 (−1.01–0.12)	0.122
NRS 2002 score 5	−0.64 (−1.31 to 0.03)	0.059	−0.87 (−1.49 to −0.26)	**0.006**
NRS 2002 score 6	−0.16 (−1.26 to 0.94)	0.779	−1.02 (−2.04 to −0.01)	**0.047**
**Food intake**				
Mean protein intake [g], per 10 g protein	0.21 (0.11–0.32)	**<** **0.001**	0.25 (0.15–0.34)	**<** **0.001**
Mean energy intake [kcal], per 100 kcal	0.06 (0.02–0.1)	**0.002**	0.07 (0.04–0.11)	**<** **0.001**
**Laboratory markers**				
Albumin at admission [g/L]	−0.23 (−0.27 to −0.19)	**<** **0.001**	−0.29 (−0.34 to −0.25)	**<** **0.001**
CRP at admission [mg/L], per 10 mg/l	0.02 (−0.01–0.05)	0.275	−0.09 (−0.12 to −0.05)	**<** **0.001**
**Admission diagnosis**				
Infection	1.52 (0.98–2.05)	**<** **0.001**	1.51 (0.98–2.05)	**<** **0.001**
Cancer	−1.04 (−1.62 to −0.47)	**<** **0.001**	−1.22 (−1.74 to −0.7)	**<** **0.001**
Cardiovascular disease	0.28 (−0.49 to 1.04)	0.476	0.77 (0.05–1.48)	**0.035**
Failure to thrive	−1.76 (−2.73 to −0.8)	**<** **0.001**	−1.03 (−1.94 to −0.13)	**0.025**
Lung disease	0.03 (−1.07 to 1.13)	0.958	0.56 (−0.44 to 1.57)	0.272
Gastrointestinal disease	−0.97 (−1.87 to −0.07)	**0.035**	−1.53 (−2.36 to −0.71)	**<** **0.001**
**Comorbidities**				
Malignant disease	−0.62 (−1.13 to −0.12)	**0.016**	−0.95 (−1.42 to −0.49)	**<** **0.001**
Chronic kidney disease	0.33 (−0.19 to 0.84)	0.213	0.34 (−0.13 to 0.81)	0.151
Coronary heart disease	−0.06 (−0.64 to 0.51)	0.829	0.21 (−0.32 to 0.73)	0.438
Diabetes	−0.25 (−0.83 to 0.33)	0.394	−0.39 (−0.91 to 0.14)	0.151
Congestive heart failure	0.45 (−0.18 to 1.08)	0.159	0.66 (0.09–1.23)	**0.024**

*CI 95%* Confidence interval, *Coeff* Coefficient.

Bold values indicates statistical significant *P*-values (*P* < 0.05).

### Effect of nutritional intervention in association with kinetics of serum albumin levels

**Fig. 3:**
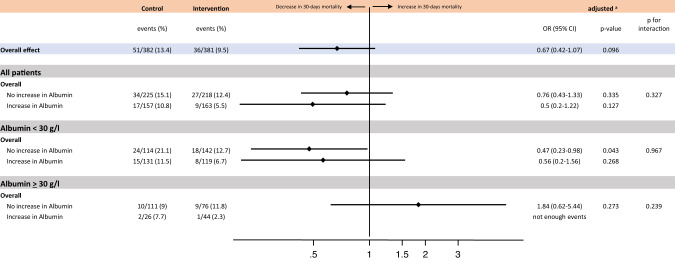
Forest plot for 30-days mortality, Effect of nutritional intervention depending on kinetics of serum albumin concentrations. OR Odds ratio, 95% CI Confidence interval. ^a^ adjusted for age, sex, main diagnosis, comorbidities and study center. Values are presented on a logarithmic scale for better visualization.

Last, we evaluated whether the effectiveness of nutritional support concerning 30-day mortality in the intervention group and control group would differ according to changes in albumin concentrations. The mortality benefit of nutritional support was independent of changes in albumin concentrations in the overall population (p interaction 0.327), and also when stratified by albumin concentrations (Fig. 3).

## Discussion

In this secondary post-hoc analysis of a randomized clinical trial, we investigated first whether nutritional support affects short-term changes in serum albumin concentrations among medical inpatients at nutritional risk, and second, whether an increase in albumin concentrations has prognostic implications regarding clinical outcome and treatment response. We found that 42% of patients in our study population had an increase in albumin concentrations from baseline to day 7 and, compared with usual hospital food, nutritional support was not associated with a more pronounced increase in albumin concentrations. The changes in serum albumin concentrations, however, provided prognostic information, and mortality and length of hospital stay were significantly lower in patients with an increase in albumin concentration compared with those with decrease. Finally, patients with and without increase in albumin concentrations had a similar treatment response to the nutritional intervention and monitoring of albumin from baseline to day 7 was, thus, not helpful for predicting response to nutritional support.

These findings are largely in line with a recent statement by the American Society for Parenteral and Enteral Nutrition (ASPEN) stating that “visceral proteins have not been shown to be sensitive markers of energy and protein intake adequacy and therefore should not be a guide for therapeutic changes.” [2] We observed that protein and energy intake was associated with increase in albumin concentration in the univariate model but not the multivariate model in this study. This may be due to direct effects of nutrition on albumin concentration and secondary effects as an improvement in appetite with leading to more food intake is also typically seen when the underlying disease improves. Thus, higher intake as a surrogate for better health, may have confounded the analysis.

While nutritional intervention did not show differences in albumin concentrations in the overall population suggesting that nutritional support had little effect on the short-term changes in serum albumin concentrations over one week, there were some significant effects in the subgroup of patients in participants with a high *vs*. normal albumin concentration at baseline ( ≥ 30 g/L) and low levels of the inflammatory marker CRP. We have previously reported that inflammation as assessed by CRP is an important predictor for the effect of nutrition on health outcomes and patients with low inflammation had most benefits [27]. About 25% of these patients had inflammation due to an infection, and the increase in albumin concentration over time in these patients may be due to the resolution of inflammation with subsequent increases in albumin concentrations, and rather than nutritional effects.

Interestingly, most patients in our study showed an absolute decrease in serum albumin concentrations in the short-term follow-up over 7 days. We assume that this was due to the acute disease of our patients with an increase in catabolism and the fact that human serum albumin has a half-life of about 19 days [34]. Therefore, the 7-day-course of our analysis was too short and longer-term follow-up may show an increase in concentrations at a later time point when the acute disease and inflammation has resolved. It would have been interesting to also look into prealbumin kinetics over 7 days for comparison, which, however was not possible due to missing day 7 data for prealbumin [28].

We are aware of several strengths and limitations. To our knowledge, this analysis is among the first and maybe the most comprehensive study to look at changes in serum albumin concentrations in a large population of medical patients from a previous randomized trial with detailed information about nutritional intake and the resolution of the disease. In the 1980s Winkler et al. [35]. as well as Ota et al. [36]. found prealbumin to be a better indicator for response to short-term nutritional support than other visceral proteins including albumin, due to the shorter half-life, in patients undergoing surgery and those with cancer, respectively. Since then, most studies focused on prealbumin and other visceral proteins as monitoring-parameters for nutritional therapy and little attention has been paid to albumin [37]. Unfortunately, we did not measure prealbumin concentrations over time, but only had admission levels measured in a subset of patients [28]. Thus, it is not possible within this analysis to compare albumin to prealbumin regarding prognostic implications. Other important limitations include possible selection bias due to the underlying trial and because only patients with two albumin concentrations (baseline and day 7) were included. There is also risk for residual confounding although we did adjust our analysis for important confounders. Thus, we excluded some patients who died within the first week or were discharged home early. Also, with 763 patients, our study is larger compared to previous trials but may still be underpowered to find small differences in clinical response in patients with low and normal prealbumin levels. Overall, as a secondary analysis, our findings are hypothesis-generating and need validation in prospective studies.

## Conclusion

Results from this secondary analysis including medical inpatients at nutritional risk indicate that nutritional support did not increase concentrations of albumin within 7 days, and changes in serum albumin concentrations did not correlate with treatment response to nutritional interventions. However, an increase in albumin concentrations possibly mirroring resolution of inflammation was associated with better clinical outcomes. Repeated in-hospital albumin measurements in the short-term is, thus, not indicated for monitoring of patients receiving nutritional support but provides prognostic information.

### Supplementary information

Supplementary analysis

## Data Availability

We intend to make data collected for the study, including anonymized individual participant data and a data dictionary defining each field in the set, available to others. Related documents will be available, including the trial protocol and the statistical analysis plan. These data will be available with the publication of our main manuscript and all secondary projects as outlined in our trial protocol on receipt of a letter of intention detailing the study hypothesis and statistical analysis plan. The steering committee of this trial will discuss all requests and decide on the basis of the scientific rigor of the proposal whether data sharing is appropriate. All applicants are asked to sign a data access agreement. Please send any request to the principal investigator of this trial.
