# Supplementary material for: Changes in serum albumin concentrations over 7 days in medical inpatients with and without nutritional support. A secondary post-hoc analysis of a randomized clinical trial
Source: Eur J Clin Nutr. 2023 Jul 7;77(10):989–97. doi: 10.1038/s41430-023-01303-w (PMC10564620; doi:10.1038/s41430-023-01303-w)
Supplement: Supplementary file 1 — Supplementary analysis [file 41430_2023_1303_MOESM1_ESM.pdf]

Supplementary Material

Changes in serum albumin concentrations over 7 days in medical inpatients with and without nutritional support.

A secondary post-hoc analysis of a randomized clinical trial

Fabienne Boesiger[1]*; Alessia Poggioli[1]*, Claudine Netzhammer[1]*, Céline Bretscher[1], MD; Nina Kaegi-Braun[1], MD; Pascal Tribolet[2], Carla Gressies[1], Alexander Kutz[1], Dileep N. Lobo[5,6], Zeno Stanga[3], MD, Beat Mueller[1,4], Philipp Schuetz[1,4] on behalf of the EFFORT team

*equally contributing first authors

[1] Medical University Department, Division of General Internal and Emergency Medicine, Kantonsspital Aarau, Aarau, Switzerland

[2] Department of Health Professions, Bern University of Applied Sciences, Bern, Switzerland and Faculty of Life Sciences, University of Vienna, Vienna, Austria

[3] Division of Diabetes, Endocrinology, Nutritional Medicine, and Metabolism, Inselspital, Bern University Hospital, University of Bern, Bern, Switzerland

[4] Department of Clinical Research, University Hospital Basel, University of Basel, Basel, Switzerland.

[5] Gastrointestinal Surgery, Nottingham Digestive Diseases Centre and National Institute for Health Research (NIHR) Nottingham Biomedical Research Centre, Nottingham University Hospitals NHS Trust and University of Nottingham, Queen's Medical Centre, Nottingham, UK

6 MRC Versus Arthritis Centre for Musculoskeletal Ageing Research, School of Life Sciences, University of Nottingham, Queen’s Medical Centre, Nottingham, UK

Corresponding author: Philipp Schuetz, MD, MPH, Medical University Department, University of Basel, Kantonsspital Aarau, Tellstrasse 25, CH-5001 Aarau, Switzerland (philipp.schuetz@ksa.ch)

NRS 2002

The Nutritional Risk Screening Score (NRS 2002) is composed of two main parts: impaired nutritional status and severity of disease (≈ stress metabolism), each with a scoring system from 0 (absent) to 3 (severe). Mild impaired nutritional status (Score 1) was defined as weight loss > 5% in 3 months or food intake below 50-75% of normal requirement in the preceding week. Moderate impaired nutritional status (Score 2) was defined as weight loss > 5% in 2 months or BMI < 18.5-20.5 kg/m2 plus impaired general condition or food intake of 25-75% of normal requirements in the preceding week. A severe impaired nutritional status means weight loss > 5% in 1 month and weight loss > 15% in 3 months respectively or BMI < 18.5 kg/m2 plus impaired general condition or food intake of 0-25% of requirements in the preceding week. A mild severity of disease (Score 1) was defined as e.g., hip fracture, chronic patients, in particular with acute complications such as cirrhosis or COPD, patients with chronic hemodialysis, diabetes or an oncologic disease. Major abdominal surgery, stroke, severe pneumonia or hematologic malignancy were rated as a moderate severity of disease (Score 2). Head injury, bone marrow transplantation and need of intensive care were considered as severe. This list lacks of completeness. The two scores combined add up to the total score. One has to add 1 score to the total score if age ≥ 70 years to correct for frailty of elderly. An age-corrected total score ≥ 3 indicates “nutritionally at risk” and a nutritional support should be considered.[1]

Primary endpoints

- For the prognostic and predictive analyses: 30-day mortality; defined as mortality of all causes from inclusion to day 30 [2,3]
- For the prognostic analysis only 180-day mortality; defined as mortality of all causes from inclusion to day 180 [2,3]
-

Secondary endpoints:

- Adverse clinical outcome within 30 days:
  I. 30-day all-cause mortality: defined as mortality of all causes from inclusion to day 30 [2,3]
  II. Admission to ICU: defined as transfer to the intensive care unit from hospital ward from admission to day 30 [2,3]
  III. Non-elective hospital readmission after discharge: defined as not intended readmission to hospital after discharge from the index hospitalisation to day 30 [2,3]
  IV. Major complications: defined as one of the following events from inclusion to day 30: [2,3]
    - Nosocomial infection or abscess
    - Respiratory failure with requiring ventilation (invasive or non-invasive)
    - Major cardiovascular event including cardiac arrest, myocardial infarction, stroke, intracranial bleeding and pulmonary embolism

- Acute renal failure (two times increase of baseline creatinine or new requirement of dialysis, eg, due to volume overload or electrolyte disturbance)
- Gastro-intestinal failure including haemorrhage, intestinal perforation and acute pancreatitis

V. Decline in functional status: defined as a decline in Barthel's index of 10% or more from inclusion to day 30
Barthel's index is measuring tool for performance of activities of daily living including feeding, personal hygiene (bathing/showering, grooming), dressing, bladder and bowel control, toilet use, transferring from bed to chair and back, mobility on surface level and stair climbing. Barthel's index results in a score from 100 to 0 with higher lower scores indication more severe disability. [2-4]

- Length of hospital stay: defined as total length of hospitalisation during the index hospital stay from inclusion to day 30 [2,3]
- Loss of function: defined as a decline in Barthel's index of 10% or more from inclusion to day 30 [2-4]
- Quality of life after 180 days: measured on admission and on day 180 on the basis of the EuroQol Group 5-Dimensions Self-Report Questionnaire including the European Quality of life 5 Dimensions index and the visual-analogue scale (EQ5D-VAS).
European Quality of life 5 Dimensions index includes questions about mobility, self-care, usual activities (ie, work, study, housework, family or leisure activities), pain/discomfort, anxiety/depression and current health state compared to the level of health over the past 12 months) resulting in scores from 0 to 1, with lower scores indication worse life quality.
The visual-analogue scale ranks the current health state in a score from 0 to 100 with higher scores indication better health state. [2,3,5]

References:

1. Kondrup J. Nutritional risk screening (NRS 2002): a new method based on an analysis of controlled clinical trials. *Clinical Nutrition.* 2003;22(3):321-336.

2. Schuetz P, Fehr R, Baechli V, et al. Individualised nutritional support in medical inpatients at nutritional risk: a randomised clinical trial. *Lancet.* Jun 8 2019;393(10188):2312-2321.

3. Schuetz P, Fehr R, Baechli V, et al. Design and rationale of the effect of early nutritional therapy on frailty, functional outcomes and recovery of malnourished medical inpatients trial (EFFORT): a pragmatic, multicenter, randomized-controlled trial. *International Journal of Clinical Trials.* 2018;5(3).

4. Mahoney FI, Barthel DW. Functional Evaluation: The Barthel Index. *Md State Med J.* Feb 1965;14:61-65.

5. Brooks R. EuroQol: the current state of play. *Health Policy.* Jul 1996;37(1):53-72.

Table 1: Baseline characteristics, additionally stratified by low C-reactive protein (< 100 g/L) and high C-reactive protein (≥ 100 g/L) at admission

	CRP < 100 mg/l					CRP ≥ 100 mg/l		
	Overall	Increase in Albumin after 7 days	No Increase in Albumin after 7 days	p-value	Overall	Increase in Albumin after 7 days	No Increase in Albumin after 7 days	p-value
n	511	213	298		252	107	145	
**Sociodemographics**								
Age, mean (SD) years	74.1 (12.6)	74.0 (13.4)	74.2 (12.0)	0.86	71.6 (13.3)	71.7 (13.8)	71.5 (13.0)	0.93
Male sex	268 (52.4%)	104 (48.8%)	164 (55.0%)	0.17	141 (56.0%)	60 (56.1%)	81 (55.9%)	0.97
**Nutritional assessment**								
BMI, mean (SD) kg/m2	24.6 (5.1)	25.1 (5.7)	24.3 (4.7)	0.089	25.4 (5.6)	25.8 (5.1)	25.1 (5.9)	0.31
Weight at admission, mean (SD) kg	71.0 (16.0)	72.9 (17.8)	69.6 (14.3)	0.052	73.9 (16.9)	73.7 (15.8)	74.1 (17.7)	0.88
Height, mean (SD) cm	167.7 (8.7)	167.4 (9.3)	168.0 (8.3)	0.40	168.9 (9.3)	168.1 (9.4)	169.5 (9.2)	0.24
**NRS 2002 score**								
3	157 (30.7%)	69 (32.4%)	88 (29.5%)	0.19	46 (18.3%)	17 (15.9%)	29 (20.0%)	0.77
4	200 (39.1%)	83 (39.0%)	117 (39.3%)		105 (41.7%)	45 (42.1%)	60 (41.4%)	
5	130 (25.4%)	47 (22.1%)	83 (27.9%)		78 (31.0%)	36 (33.6%)	42 (29.0%)	
6	24 (4.7%)	14 (6.6%)	10 (3.4%)		23 (9.1%)	9 (8.4%)	14 (9.7%)	
**Admission diagnosis**								
Infection	87 (17.0%)	51 (23.9%)	36 (12.1%)	**<0.001**	130 (51.6%)	68 (63.6%)	62 (42.8%)	**0.001**
Cancer	117 (22.9%)	34 (16.0%)	83 (27.9%)	**0.002**	63 (25.0%)	20 (18.7%)	43 (29.7%)	**0.047**
Cardiovascular disease	87 (17.0%)	41 (19.2%)	46 (15.4%)	0.26	2 (0.8%)	0 (0.0%)	2 (1.4%)	0.22
Failure to thrive	38 (7.4%)	10 (4.7%)	28 (9.4%)	**0.046**	14 (5.6%)	4 (3.7%)	10 (6.9%)	0.28
Lung disease	28 (5.5%)	12 (5.6%)	16 (5.4%)	0.90	12 (4.8%)	5 (4.7%)	7 (4.8%)	0.95
Gastrointestinal disease	51 (10.0%)	21 (9.9%)	30 (10.1%)	0.94	11 (4.4%)	1 (0.9%)	10 (6.9%)	**0.022**
Neurological disease	13 (2.5%)	6 (2.8%)	7 (2.3%)	0.74	1 (0.4%)	1 (0.9%)	0 (0.0%)	0.24
Renal disease	34 (6.7%)	12 (5.6%)	22 (7.4%)	0.43	5 (2.0%)	1 (0.9%)	4 (2.8%)	0.30
Metabolic disease	26 (5.1%)	11 (5.2%)	15 (5.0%)	0.95	2 (0.8%)	1 (0.9%)	1 (0.7%)	0.83
Other	18 (3.5%)	11 (5.2%)	7 (2.3%)	0.089	7 (2.8%)	2 (1.9%)	5 (3.4%)	0.45

**Comorbidities**								
Hypertension	291 (56.9%)	128 (60.1%)	163 (54.7%)	0.22	146 (57.9%)	60 (56.1%)	86 (59.3%)	0.61
Malignant disease	171 (33.5%)	61 (28.6%)	110 (36.9%)	0.051	116 (46.0%)	46 (43.0%)	70 (48.3%)	0.41
Chronic kidney disease	192 (37.6%)	87 (40.8%)	105 (35.2%)	0.20	76 (30.2%)	39 (36.4%)	37 (25.5%)	0.062
Coronary heart disease	133 (26.0%)	57 (26.8%)	76 (25.5%)	0.75	51 (20.2%)	20 (18.7%)	31 (21.4%)	0.60
Diabetes	120 (23.5%)	43 (20.2%)	77 (25.8%)	0.14	59 (23.4%)	24 (22.4%)	35 (24.1%)	0.75
Congestive heart failure	111 (21.7%)	56 (26.3%)	55 (18.5%)	**0.034**	34 (13.5%)	14 (13.1%)	20 (13.8%)	0.87
Chronic obstructive pulmonary disease	62 (12.1%)	31 (14.6%)	31 (10.4%)	0.16	31 (12.3%)	10 (9.3%)	21 (14.5%)	0.22
Peripheral arterial disease	54 (10.6%)	21 (9.9%)	33 (11.1%)	0.66	19 (7.5%)	10 (9.3%)	9 (6.2%)	0.35
Cerebrovascular disease	47 (9.2%)	21 (9.9%)	26 (8.7%)	0.66	23 (9.1%)	7 (6.5%)	16 (11.0%)	0.22
Dementia	20 (3.9%)	9 (4.2%)	11 (3.7%)	0.76	3 (1.2%)	0 (0.0%)	3 (2.1%)	0.13

Legend Table 1: Abbreviations: BMI, body mass index (weight in kilograms divided by height in meters squared); NRS, Nutritional risk screening; SD, standard deviation

Table 2.1: Kinetics of serum albumin levels from baseline to day 7 and association with nutritional intervention, patients with admission c-reactive protein < 100 g/L

All patients	CRP < 100 mg/l					
			unadjusted		adjusted [a]	
	Control	Intervention	OR or Coeff (CI 95%)	p-value	OR or Coeff (CI 95%)	p-value
	n (%) or mean (SD or CI 95%)	n (%) or mean (SD or CI 95%)				
**Change in albumin after 7 days**						
**Baseline Albumin (g/L)**	30.05 (5.46)	28.88 (5.16)				
**Albumin after 7 days (g/L)**	28.96 (4.77)	28.31 (5.11)				
**Change in Albumin (g/L)**	-1.09 (-1.53--0.65)	-0.57 (-0.96--0.17)	0.3 (-0.07-1.11)	0.082	0.5 (-0.07-1.08)	0.084
**Increase vs. No Increase in albumin after 7 days**						
**No Increase in Albumin after 7 days**	155/246 (63)	143/265 (54)	reference		reference	
**Increase in Albumin after 7 days**	91/246 (37)	122/265 (46)	1.45 (1.02-2.07)	**0.039**	1.49 (1.03-2.16)	**0.035**
**Baseline-Albumin < 30 g/l**	**CRP < 100 mg/l**					
			**unadjusted**		**adjusted [a]**	
	**Control**	**Intervention**	**OR or Coeff (CI 95%)**	**p-value**	**OR or Coeff (CI 95%)**	**p-value**
	n (%) or mean (SD or CI 95%)	n (%) or mean (SD or CI 95%)				
**Change in albumin after 7 days**						
**Baseline Albumin (g/L)**	25.63 (3.01)	25.62 (3.42)				
**Albumin after 7 days (g/L)**	26.06 (3.91)	25.61 (4.17)				
**Change in Albumin (g/L)**	0.43 (-0.09-0.95)	-0.01 (-0.48-0.46)	-0.44 (-1.14-0.26)	0.215	-0.34 (-1-0.32)	0.316

**Increase vs. No Increase in albumin after 7 days**						
**No Increase in Albumin after 7 days**	56/123 (45.5)	80/159 (50.3)	reference		reference	
**Increase in Albumin after 7 days**	67/123 (54.5)	79/159 (49.7)	0.83 (0.51-1.32)	0.425	0.85 (0.51-1.41)	0.525
**Baseline-Albumin $\geq$ 30 g/l**	**CRP < 100 mg/l**					
			**unadjusted**		**adjusted [a]**	
	**Control**	**Intervention**	**OR or Coeff (CI 95%)**	**p-value**	**OR or Coeff (CI 95%)**	**p-value**
	n (%) or mean (SD or CI 95%)	n (%) or mean (SD or CI 95%)				
**Change in albumin after 7 days**						
**Baseline Albumin (g/L)**	34.46 (3.39)	33.77 (3.02)				
**Albumin after 7 days (g/L)**	31.85 (3.67)	32.37 (3.42)				
**Change in Albumin (g/L)**	-2.61 (-3.23--2)	-1.4 (-2.07--0.73)	1.21 (0.31-2.12)	**0.009**	1.17 (0.24-2.1)	**0.014**
**Increase vs. No Increase in albumin after 7 days**						
**No Increase in Albumin after 7 days**	99/123 (80.5)	63/106 (59.4)	reference		reference	
**Increase in Albumin after 7 days**	24/123 (19.5)	43/106 (40.6)	2.82 (1.56-5.08)	**0.001**	2.78 (1.49-5.17)	**0.001**

Legend Table 2.1: Abbreviations: SD, standard deviation; CI 95%, confidence interval; OR, odds ratio; Coeff, Coefficient;

[a] adjusted for age, sex, diagnoses, center

Table 2.2: Kinetics of serum albumin levels from baseline to day 7 and association with nutritional intervention, patients with admission c-reactive protein ≥ 100 g/L

All patients	CRP ≥ 100 mg/l					
			unadjusted		adjusted [a]	
	Control	Intervention	OR or Coeff (CI 95%)	p-value	OR or Coeff (CI 95%)	p-value
	n (%) or mean (SD or CI 95%)	n (%) or mean (SD or CI 95%)				
**Change in albumin after 7 days**						
**Baseline Albumin (g/L)**	24.11 (5.08)	24.9 (4.67)				
**Albumin after 7 days (g/L)**	24.06 (5.08)	23.63 (4.67)				
**Change in Albumin (g/L)**	-0.06 (-0.63-0.52)	-1.26 (-1.95--0.58)	-1.21 (-2.09--0.33)	**0.007**	-0.99 (-1.88--0.11)	**0.028**
**Increase vs. No Increase in albumin after 7 days**						
**No Increase in Albumin after 7 days**	70/136 (51.5)	75/116 (64.7)	reference		reference	
**Increase in Albumin after 7 days**	66/136 (48.5)	41/116 (35.3)	0.58 (0.35-0.96)	**0.035**	0.63 (0.37-1.08)	0.091

Baseline-Albumin < 30 g/l	CRP ≥ 100 mg/l					
			unadjusted		adjusted [a]	
	Control	Intervention	OR or Coeff (CI 95%)	p-value	OR or Coeff (CI 95%)	p-value
	n (%) or mean (SD or CI 95%)	n (%) or mean (SD or CI 95%)				
**Change in albumin after 7 days**						
**Baseline Albumin (g/L)**	23.05 (3.97)	23.7 (3.45)				
**Albumin after 7 days (g/L)**	23.35 (4.61)	23 (4.36)				
**Change in Albumin (g/L)**	0.3 (-0.28-0.89)	-0.7 (-1.33--0.08)	-1.01 (-1.86--0.16)	**0.021**	-0.71 (-1.56-0.14)	0.101

**Increase vs. No Increase in albumin after 7 days**						
**No Increase in Albumin after 7 days**	58/122 (47.5)	62/102 (60.8)	reference		reference	
**Increase in Albumin after 7 days**	64/122 (52.5)	40/102 (39.2)	0.58 (0.34-1)	**0.048**	0.63 (0.36-1.11)	0.111
**Baseline-Albumin ≥ 30 g/l**	**CRP ≥ 100 mg/l**					
			**unadjusted**		**adjusted [a]**	
	**Control**	**Intervention**	**OR or Coeff (CI 95%)**	**p-value**	**OR or Coeff (CI 95%)**	**p-value**
	n (%) or mean (SD or CI 95%)	n (%) or mean (SD or CI 95%)				
**Change in albumin after 7 days**						
**Baseline Albumin (g/L)**	33.41 (4.18)	33.59 (2.81)				
**Albumin after 7 days (g/L)**	30.21 (0)	28.24 (4.39)				
**Change in Albumin (g/L)**	-3.21 (-4.91--1.5)	-0.7 (-1.33--0.08)	-2.15 (-5.18-0.88)	0.156	-0.48 (-5.07-4.11)	0.828
**Increase vs. No Increase in albumin after 7 days**						
**No Increase in Albumin after 7 days**	12/14 (85.7)	13/14 (92.9)	reference		reference	
**Increase in Albumin after 7 days**	2/14 (14.3)	1/14 (7.1)	0.46 (0.04-5.77)	0.548	0.46 (0.04-5.77)	0.548

Legend Table 2.2: Abbreviations: SD, standard deviation; CI 95%, confidence interval; OR, odds ratio; Coeff, Coefficient;

[a] adjusted for age, sex, diagnoses, center

Table 3.1: Clinical and functional outcomes depending on kinetics of serum albumin levels from baseline to day 7, overall study population

	**Overall**				
	events	**unadjusted**		**adjusted [a]**	
	n (%) or mean (SD)	OR or Coeff (95% CI)	p-value	OR or Coeff (95% CI)	p-value
**Primary endpoint**					
**180-day mortality**					
No Increase in Albumin	158/443 (35.7)	reference		reference	
Increase in Albumin	74/320 (23.1)	0.54 (0.39-0.75)	**<0.001**	0.63 (0.44-0.9)	**0.012**
**Secondary endpoints**					
**Adverse Outcome within 30 days**					
No Increase in Albumin	138/443 (31.2)	reference		reference	
Increase in Albumin	75/320 (23.4)	0.68 (0.49-0.94)	**0.019**	0.72 (0.51-1.02)	0.068
**Length of hospital stay**					
No Increase in Albumin	11.16(7.3)	reference		reference	
Increase in Albumin	8.8(5.6)	-2.35 (-3.31--1.39)	**<0.001**	-2.16 (-3.14--1.18)	**<0.001**
**Loss of function (Barthel index)**					
No Increase in Albumin	83/443 (18.7)	reference		reference	
Increase in Albumin	39/320 (12.2)	0.6 (0.4-0.91)	**0.016**	0.66 (0.42-1.02)	0.058
**Quality of life (EQ5D-VAS)**					
No Increase in Albumin	68(19.7)	reference		reference	
Increase in Albumin	68.01(19.6)	0.01 (-3.59-3.62)	0.994	-0.43 (-4.1-3.24)	0.817

Legend Table 3.1: Abbreviations: SD, standard deviation; CI 95%, confidence interval; OR, odds ratio; Coeff, Coefficient.

[a] adjusted for age, sex, diagnoses, center

Table 3.2: Clinical and functional outcomes depending on kinetics of serum albumin levels from baseline to day 7, patients with admission c-reactive protein < 100 g/L

	**CRP < 100 mg/l**				
	events	**unadjusted**		**adjusted** [a]	
	n (%) or mean (SD)	OR or Coeff (95% CI)	p-value	OR or Coeff (95% CI)	p-value
**Primary endpoint**					
**180-day mortality**					
No Increase in Albumin	101/298 (33.9)	reference		reference	
Increase in Albumin	49/213 (23)	0.58 (0.39-0.87)	**0.008**	0.68 (0.43-1.07)	0.096
**Secondary endpoints**					
**Adverse Outcome within 30 days**					
No Increase in Albumin	90/298 (30.2)	reference		reference	
Increase in Albumin	47/213 (22.1)	0.65 (0.44-0.98)	**0.041**	0.66 (0.42-1.02)	0.061
**Length of hospital stay**					
No Increase in Albumin	10.57(6.8)	reference		reference	
Increase in Albumin	8.93(5.7)	-1.64 (-2.76--0.52)	**0.004**	-1.35 (-2.5--0.2)	**0.022**
**Loss of function (Barthel index)**					
No Increase in Albumin	53/298 (17.8)	reference		reference	
Increase in Albumin	22/213 (10.3)	0.53 (0.31-0.91)	**0.02**	0.54 (0.31-0.96)	**0.036**
**Quality of life (EQ5D-VAS)**					
No Increase in Albumin	68.17(19.7)	reference		reference	
Increase in Albumin	65.97(18.7)	-2.21 (-6.48-2.06)	0.31	-2.8 (-7.2-1.6)	0.211

Legend Table 3.2: Abbreviations: SD, standard deviation; CI 95%, confidence interval; OR, odds ratio; Coeff, Coefficient.

[a] adjusted for age, sex, diagnoses, center

Table 3.3: Clinical and functional outcomes depending on kinetics of serum albumin levels from baseline to day 7, patients with admission c-reactive protein ≥ 100 g/L

	CRP ≥ 100 mg/l				
	events	**unadjusted**		**adjusted** [a]	
	n (%) or mean (SD)	OR or Coeff (95% CI)	p-value	OR or Coeff (95% CI)	p-value
**Primary endpoint**					
**180-day mortality**					
No Increase in Albumin	57/145 (39.3)	reference		reference	
Increase in Albumin	25/107 (23.4)	0.47 (0.27-0.82)	**0.008**	0.5 (0.26-0.96)	**0.037**
**Secondary endpoints**					
**Adverse Outcome within 30 days**					
No Increase in Albumin	48/145 (33.1)	reference		reference	
Increase in Albumin	28/107 (26.2)	0.72 (0.41-1.24)	0.237	0.8 (0.44-1.46)	0.472
**Length of hospital stay**					
No Increase in Albumin	12.35(8.3)	reference		reference	
Increase in Albumin	8.55(5.4)	-3.8 (-5.61--1.99)	**<0.001**	-3.62 (-5.47--1.77)	**<0.001**
**Loss of function (Barthel index)**					
No Increase in Albumin	30/145 (20.7)	reference		reference	
Increase in Albumin	17/107 (15.9)	0.72 (0.38-1.4)	0.335	0.86 (0.42-1.76)	0.674
**Quality of life (EQ5D-VAS)**					
No Increase in Albumin	67.6(19.8)	reference		reference	
Increase in Albumin	72.25(20.8)	4.65 (-2.03-11.33)	0.171	5.28 (-1.44-12)	0.123

Legend Table 3.3: Abbreviations: SD, standard deviation; CI 95%, confidence interval; OR, odds ratio; Coeff, Coefficient.

[a] adjusted for age, sex, diagnoses, center
